# Early resuscitation priorities in two-provider EMS crews: an interpretive commentary

**DOI:** 10.1186/s12873-026-01549-0

**Published:** 2026-04-10

**Authors:** Roman Sýkora, Jiří Chvojka, Nikola Kukačková, Viktorie Šimečková, Jiří Smetana

**Affiliations:** 1 Emergency Medical Service of Karlovy Vary Region, Závodní 390/98C, Karlovy Vary, 36006 Czech Republic; 2 Department of Anaesthesia and Intensive Care Medicine, University Hospital Královské Vinohrady, Third Faculty of Medicine, Charles University, Prague, Czech Republic; 3 Medical College Prague, Prague, Czech Republic; 4 Emergency Medical Service of the Pilsen Region, Pilsen, Czech Republic; 5 First Department of Internal Medicine, University Hospital Pilsen, Pilsen, Czech Republic; 6 Faculty of Biomedical Engineering, Czech Technical University in Prague, Kladno, Czech Republic

**Keywords:** Out-of-hospital cardiac arrest, Emergency medical services, Two-provider crews, Early resuscitation, Task prioritization, Guideline implementation, Cognitive workload, Limited resources, Prehospital care

## Abstract

**Background:**

International resuscitation guidelines are grounded in physiological principles and outcome-based evidence, but they implicitly assume staffing levels that allow multiple time-critical interventions to be delivered in parallel. In many emergency medical systems, however, advanced life support is initially provided by a two-provider crew working alone for a substantial period. Under these conditions, the parallel task execution described in guidelines may become structurally impossible because of capacity constraints. This commentary therefore focuses on adult non-traumatic out-of-hospital cardiac arrest managed according to standard adult ALS guidelines.

**Interpretive framework:**

This article is presented as an interpretive commentary and is intended to provide an explanatory perspective on how early guideline-consistent care is enacted under conditions of limited personnel and cognitive capacity, rather than as a prescriptive care algorithm. It applies exclusively to the initial, transient phase of out-of-hospital resuscitation in which advanced life support is delivered by a two-provider professional crew working alone. Its applicability ends once additional trained personnel arrive, stable task allocation becomes possible, or the initial defibrillation cycles have been completed. Deviations from ideal task sequencing are conceptualised as forced deviations, an emergent property of system-level capacity limits rather than individual performance failure. Resuscitation actions are therefore considered in terms of their relative short-term physiological penalty when delayed or interrupted: (1) critical perfusion-dependent actions, where interruption is associated with immediate physiological harm; (2) actions with lower short-term physiological penalty when briefly deferred; and (3) bounded uncertainty domains, particularly ventilation, where physiological effects are non-linear and context dependent.

**Conclusion:**

This interpretive commentary does not modify existing resuscitation guidelines. Instead, it clarifies how their physiological intent may be understood and preserved during the earliest phase of two-provider advanced life support, when parallel task execution is not feasible. By providing a shared interpretive model for early resuscitation under capacity constraint, this approach may help reduce harmful variability, cognitive overload, and misinterpretation of performance at a system level in real-world emergency medical systems.

## Background: resuscitation guidelines and operational capacity

High-quality cardiopulmonary resuscitation depends on early chest compressions, minimisation of hands-off time, timely defibrillation in shockable rhythms, and controlled ventilation [1]. These priorities are consistent across major international recommendations.

What all guidelines address less explicitly is the operational context in which these actions must be delivered. In many systems, professional resuscitation begins with a two-provider crew. Additional responders may arrive later, assist intermittently, or lack predefined roles. During this early phase, tasks described as concurrent in guidelines must be executed sequentially, competing for the same limited hands and attention. This creates a structural mismatch between guideline assumptions and operational reality at the point of care. Evidence directly examining two-provider advanced life support remains limited and indirect [2]. Early out-of-hospital resuscitation is dynamic and context dependent, making controlled trials with neurological or survival endpoints difficult in this domain. Consequently, the framework presented here is grounded primarily in physiological principles and operational constraints rather than comparative outcome data. It refers primarily to adult non-traumatic out-of-hospital cardiac arrest managed according to standard adult ALS guidelines.

## Capacity limits and forced deviations

When task demand exceeds available capacity, deviation from ideal task sequencing becomes unavoidable. Such deviations arise from predictable system-level constraints rather than insufficient training, motivation, or clinical judgment. Attempts to operationalise all guideline-recommended actions concurrently under these conditions are associated with an increased risk of chest compression interruptions and delayed defibrillation, both of which are strongly associated with worse outcomes [3–5]. 

Within this framework, forced deviations (i.e., predictable system-level failure modes under capacity constraint) are not treated as errors to be eliminated, but as phenomena that require physiological interpretation to minimise harm.

## Physiological basis for prioritisation

Experimental and clinical studies consistently demonstrate the importance of coronary perfusion pressure and cerebral blood flow for defibrillation success and return of spontaneous circulation [3, 4]. Even brief interruptions in chest compressions result in rapid loss of perfusion, with incomplete recovery after resumption [5]. 

From this perspective, early resuscitation actions can be distinguished not by procedural importance, but by the immediacy and magnitude of physiological penalty associated with their interruption or delay.

This interpretive framework organises early two-provider resuscitation actions by their short-term physiological penalty during interruption or delay. It distinguishes (i) perfusion-dependent actions associated with immediate physiological harm when interrupted (e.g., compressions and defibrillation timing), (ii) actions with lower short-term physiological penalty when briefly deferred to protect perfusion-critical tasks, and (iii) bounded uncertainty domains, where intra-arrest physiological effects are non-linear and context dependent (illustrated here using ventilation during low-flow CPR).

## Actions associated with immediate physiological penalty during interruption

The examples below are not presented as justified or acceptable deviations from protocol, but as predictable failure modes that arise under severe task and cognitive overload, with direct physiological consequences. During two-provider resuscitation, several deviations are consistently associated with rapid deterioration of coronary and cerebral perfusion:

- Delayed initiation of chest compressions once cardiac arrest is recognised [6].
- Prolonged or repeated interruptions in chest compressions, including extended pre-shock pauses [5].
- Delayed defibrillation in shockable rhythms due to competing procedures [7].
- Fixation on airway procedures that increases hands-off time [8, 9].
- Ventilation extremes that acutely impair perfusion, particularly excessive ventilation [10].

## Actions with lower short-term physiological penalty when deferred

Other interventions are associated with a lower short-term physiological penalty if briefly deferred during the earliest resuscitation phase, particularly when deferral preserves continuous chest compressions and early defibrillation:

- Vascular access and drug delivery when these interrupt chest compressions [11].
- Placement of an advanced airway when repeated or prolonged attempts compete with perfusion-critical actions [8, 9].
- Complex diagnostic reasoning or protocol branching during initial resuscitation cycles [12].

## Physiological aspects of ventilation during early two-provider CPR

During cardiopulmonary resuscitation, ventilation occupies a distinct physiological domain compared with perfusion-critical interventions. Excessive ventilation increases intrathoracic pressure, reduces venous return, and lowers coronary perfusion pressure, whereas inadequate ventilation may lead to hypoxaemia and hypercapnia, with physiological consequences that are less immediate during the low-flow state of ongoing CPR [10]. 

In this context, ventilation represents a bounded uncertainty domain: a clinical domain in which physiological effects are non-linear, context dependent, and less immediately predictable under severe capacity constraints. During uninterrupted chest compressions, the short-term haemodynamic harm of excessive ventilation is well established, while the immediate physiological consequences of briefly reduced ventilation are less clearly defined and vary with arrest duration, residual oxygen reserves, and compression quality.

This concept does not reflect diagnostic uncertainty or lack of knowledge, but rather the asymmetric and time-dependent physiological impact of competing interventions when perfusion is severely limited. Contemporary reviews highlight substantial heterogeneity and limited high-quality evidence regarding optimal ventilation targets during the intra-arrest phase, particularly in the earliest cycles of resuscitation [10, 13]. 

This interpretive framework does not advocate hypoventilation or deviation from guideline-recommended ventilation targets. Instead, it provides a physiological explanation for the observed variability in early ventilation practices under two-provider capacity constraint, and clarifies why ventilation-related decisions differ fundamentally from perfusion-dependent actions during the initial phase of CPR. These considerations apply exclusively to the intra-arrest period and should not be extrapolated to post–return of spontaneous circulation care.

## Interpretation, not replacement, of guidelines

This commentary makes no claims of outcome superiority. Its contribution lies in interpreting guideline-consistent care under conditions where parallel execution is structurally impossible. All prioritisation concepts described here are derived from, and remain subordinate to, existing resuscitation guidelines. The framework does not define an alternative standard of care and should not be interpreted as permissive guidance for omitting recommended interventions when capacity allows their safe delivery. The framework refers primarily to adult non-traumatic out-of-hospital cardiac arrest and may not be directly generalisable to paediatric, traumatic, or other special-cause cardiac arrest aetiologies.

## EMS system responsibilities: policy, training, and performance oversight

Cognitive overload is an inherent feature of early two-provider resuscitation in out-of-hospital cardiac arrest, where multiple time-critical tasks compete for limited cognitive and physical resources. Under these conditions, reliance on improvised real-time prioritisation by individual crews predictably leads to variable task sequencing, including deviations with direct physiological consequences. Evidence from prehospital emergency care demonstrates substantial variability in adherence to guidelines and protocols across systems, with organisational and system-level factors identified as key determinants of practice variability rather than individual performance, even though direct contemporary data specific to two-provider advanced life support remain limited [14]. 

Early prioritisation under capacity constraint is therefore best understood as a system-level phenomenon. Emergency medical services shape how physiological priorities are interpreted and enacted through local policies, training design, and performance oversight. Cognitive science research shows that high task complexity, time pressure, and limited working memory increase susceptibility to cognitive error and reduce decision reliability, supporting the value of structured protocols, targeted training, and the externalisation of prioritisation assumptions in mitigating cognitive burden and variability during early resuscitation [12, 15]. 

## Conclusion

The initial phase of two-provider resuscitation in out-of-hospital cardiac arrest is characterised by predictable capacity constraints that preclude parallel task execution. This interpretive framework explains why early deviations from guideline-ideal task sequencing occur and clarifies their physiological implications.

The framework applies exclusively to the early, transient phase in which advanced life support is delivered by a two-provider crew working alone and ceases to apply once additional trained personnel arrive or stable task allocation becomes possible. It does not propose alternative treatment priorities and should not be interpreted as permissive guidance for omitting any guideline-recommended interventions when capacity allows.

Rather, the framework offers a shared conceptual model for preserving the physiological intent of existing resuscitation guidelines under structural constraint. At a system level, it supports an explicit, physiologically sequenced interpretation of guidelines aligned with local Emergency Medical Services configurations, with implications for policy development, training, and performance evaluation.

By making implicit prioritisation assumptions explicit, this approach may reduce harmful variability, cognitive overload, and misinterpretation of performance during the earliest phase of resuscitation in real-world emergency medical systems.

## Data Availability

Not applicable.

## Abbreviations

CPR: Cardiopulmonary Resuscitation
EMS: Emergency Medical Services
ERC: European Resuscitation Council

## Declarations

### Authors’ information

All authors are involved in professional education in resuscitation, emergency medical services, or academic medical training, and have professional experience with the development, teaching, or implementation of procedures for two-provider EMS response teams. N.K., J.C. are active instructors of the European Resuscitation Council (ERC) within the framework of the Czech Resuscitation Council. R.S. has been involved in ERC teaching activities within the Czech Resuscitation Council framework. R.S., J.C., N.K., V.Š., J.S. serve as instructors in emergency medical services (EMS) educational programmes. R.S. and J.C. are university lecturers at medical faculties, and R.S. and V.Š. teach at faculties providing paramedic education.
